# Are wearable heart rate measurements accurate to estimate aerobic energy cost during low-intensity resistance exercise?

**DOI:** 10.1371/journal.pone.0221284

**Published:** 2019-08-22

**Authors:** Victor M. Reis, Jeferson M. Vianna, Tiago M. Barbosa, Nuno Garrido, Jose Vilaça Alves, André L. Carneiro, Felipe J. Aidar, Jefferson Novaes

**Affiliations:** 1 Research Center in Sports Sciences, Health Sciences & Human Development, CIDESD, Vila Real, Portugal; 2 University of Trás-os-Montes & Alto Douro, Vila Real, Portugal; 3 Federal University at Juiz de Fora, Minas Gerais, Brazil; 4 Nanyang Technologic University, Singapore, Singapore; 5 Polytechnic Institute of Bragança, Bragança, Portugal; 6 State University at Montes Claros, Minas Gerais, Brazil; 7 Sergipe Federal University, Aracaju, Brazil; São Paulo State University (UNESP), BRAZIL

## Abstract

The aim of the present study was to assess the accuracy of heart rate to estimate energy cost during eight resistance exercises performed at low intensities: half squat, 45° inclined leg press, leg extension, horizontal bench press, 45° inclined bench press, lat pull down, triceps extension and biceps curl. 56 males (27.5 ± 4.9 years, 1.78 ± 0.06 m height, 78.67 ± 10.7 kg body mass and 11.4 ± 4.1% estimated body fat) were randomly divided into four groups of 14 subjects each. Two exercises were randomly assigned to each group and subjects performed four bouts of 4-min constant-intensity at each assigned exercise: 12%, 16%, 20% and 24% 1-RM. Exercise and intensity order were random. Each subject performed no more than 2 bouts in the same testing session. A minimum recovery of 24h was kept between sessions. During testing VO2 was measured with Cosmed K4b2 and heart rate was measured with Polar V800 monitor. Energy cost was calculated from mean VO2 during the last 30-s of each bout by using the energy equivalent 1 ml O_2_ = 5 calorie. Linear regressions with heart rate as predictor and energy cost as dependent variable were build using mean data from all subjects. Robustness of the regression lines was given by the scatter around the regression line (S*y*.*x*) and Bland-Altman plots confirmed the agreement between measured and estimated energy costs. Significance level was set at p≤0.05. The regressions between heart rate and energy cost in the eight exercises were significant (p<0.01) and robustness was: half squat (S*y*.*x* = 0,48 kcal·min^-1^), 45° inclined leg press (S*y*.*x* = 0,54 kcal·min^-1^), leg extension (S*y*.*x* = 0,59 kcal·min^-1^), horizontal bench press (S*y*.*x* = 0,47 kcal·min^-1^), 45° inclined bench press (S*y*.*x* = 0,54 kcal·min^-1^), lat pull down (S*y*.*x* = 0,28 kcal·min^-1^), triceps extension (S*y*.*x* = 0,08 kcal·min^-1^) and biceps curl (S*y*.*x* = 0,13 kcal·min^-1^). We conclude that during low-intensity resistance exercises it is possible to estimate aerobic energy cost by wearable heart rate monitors with errors below 10% in healthy young trained males.

## Introduction

Aerobic exercise intensity and its energy cost (EC) are often assessed with the use of wearable heart rate monitors. This procedure relies on the good agreement between heart rate, exercise intensity and EC in low- to moderate-intensity steady-state aerobic exercise. On the contrary, resistance exercise (RE) intensity is not often controlled by heart rate (HR), neither its energy cost is commonly estimated with heart rate measurements. Indeed, despite HR is described as a strong predictor of EC aerobic steady-state exercise such as running or cycling [1] it is rarely pointed out as an accurate predictor of energy cost during intermittent or non-steady-state exercise conditions [2,3]. Typically, a relative error below 10% in HR is warranted to consider this measure an indicator of energy cost [3].

Resistance exercise (RE), when performed at low-intensities, presents bioenergetics that are quite like those described for aerobic steady-state exercise, with a major aerobic contribution to energy release [4]. Moreover, it has been proposed that lactic threshold during RE can be located somewhere close to 30% 1-RM intensity [5,6]. If so, RE performed at intensities below that threshold are expected to be described by typical aerobic exercise bioenergetics. Before time RE has been used overtime to attain strength gains and muscle hypertrophy targets, both in sports training or aesthetics settings. Presently, RE is now included in programs which are designed to address weight loss and to target recommended energy cost values [7]. However, current evidence on rate-based energy cost measurements in isolated RE is still scarce, and especially, at low-intensity loads. Due the growing interest of low-intensity RE (i.e. to address the elderly or some pathologies) it is necessary to accumulate data on the specific energy cost of the most popular exercises and, in the future, to use such data to build technology that enables accurate calorie count during RE. [4]

The aim of the present study was to assess the accuracy of heart rate to estimate energy cost during eight resistance exercises performed at low intensities (from 12% to 24% 1-RM).

## Materials and methods

### Participants

The sample comprised a total of 56 males (27.5 ± 4.9 years, 1.78 ± 0.06 m height, 78.67 ± 10.7 kg body mass and 11.4 ± 4.1% estimated body fat) engaged in RE training for at least one year with three or more training sessions per week. They were volunteers recruited in four fitness centers as reported elsewhere [4]. Were excluded those who reported the use of drugs which could influence their cardiorespiratory response. After medical approval, the volunteers received the explanations about the procedures, as well as the risks and discomforts involved in the study and signed the written consent form. All procedures were approved by the Review Board of the Research Center in Sports Sciences, Health Sciences & Human Development and were conducted according to the Declaration of Helsinki. The volunteers were asked not to perform any strenuous exercise during the period of the experiment.

### Experimental design

The subjects were randomly divided in 4 groups with 14 participants each. Two out of the eight exercises were randomly assigned to each group. All testing was performed in the afternoon (except for the anthropometric measurements), at a temperature between 20-25C° and 35–45% relative air humidity. Each subject was submitted to six testing sessions, as follows.

In the morning of the first day, anthropometrics measures were taken (height, body mass, and five skin folds: chest, mid-axillary, tricipital, sub scapular, abdominal, supra iliac, and thigh). A calibrated caliper (Lange, Cambridge Scientific Industries, USA) and a digital medical scale with stadiometer (Seca 763, USA) were used for all measurements. Body density was calculated using the equation proposed by Jackson and Pollock [8] and Siri's equation was used to convert the density in percentage of fat mass. In the afternoon of the same day, the participants underwent a 1-RM test at the assigned exercises [9]. This same testing was repeated on the second visit (72 hours later). The highest 1-RM with less than 5% difference was considered as the true 1-RM.

During the third to the sixth visit the subjects performed four bouts of 4-min constant-intensity exercise (intensities of 12%, 16%, 20% and 24% 1-RM) in each of assigned exercise. Exercise order for exercises and for intensity were random and recovery between sessions was 48-hours. All exercises were performed with trademark standardized machines (*Panatta Sport*, Apiro, Italy). No warm-up was performed before any exercise and an electronic metronome sound established a cadence of 15 repetitions per minute (2 s on the eccentric and 2 seconds on the concentric phase), as explained elsewhere [4].

### Measurements

Expired gases were measured breath-by-breath during all exercise with a K4b2 device (*COSMED®*, Rome, Italy). To minimize respiratory artifacts and assure the accuracy of measurements, the participants were asked to avoid Valsalva maneuver [10]. Equipment was calibrated before each testing following the manufacturer's specifications [11,12]. Data was recorded in 10 s intervals and the mean oxygen uptake (VO_2_) in the last 30 s of exercise [13] in the four exercise intensities (12, 16, 20 and 24% 1-RM) was plotted against heart rate with a linear regression model. Heart rate (HR) was continuously measured with a Polar V800 device and the average HR in the last 30 s of exercise was included in the regression. Resting measurements of HR and VO_2_ were also included in the regression by a non-forced procedure. Measured O_2_ was converted into energy units (calorie) by a conversion factor of 1 ml O_2_ = 5 calorie.

### Statistical analysis

Simple linear regressions with heart rate as predictor and energy cost as dependent variable were established with mean data from the whole sample. The scatter around the regression line was used as a measure of the goodness of the fit. Bland-Altman plots were performed in order to check the agreement between measures and estimated energy cost values at the 24% 1-RM intensity. Paired t-test was used to analyze differences between measured and predicted energy cost. Significance was set at 5%.

## Results

**Fig 1:**
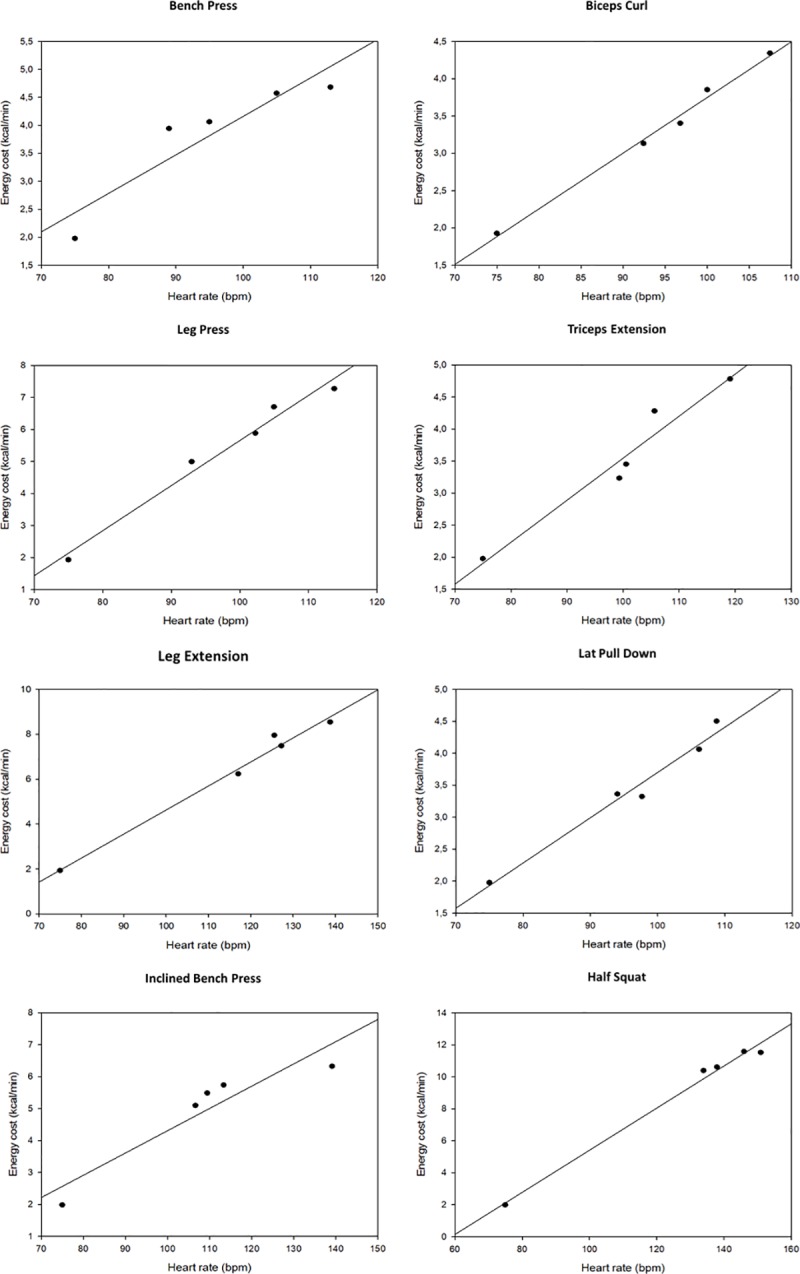
Simple linear regressions between heart rate and energy cost in the eight resistance exercises.

**Fig 2:**
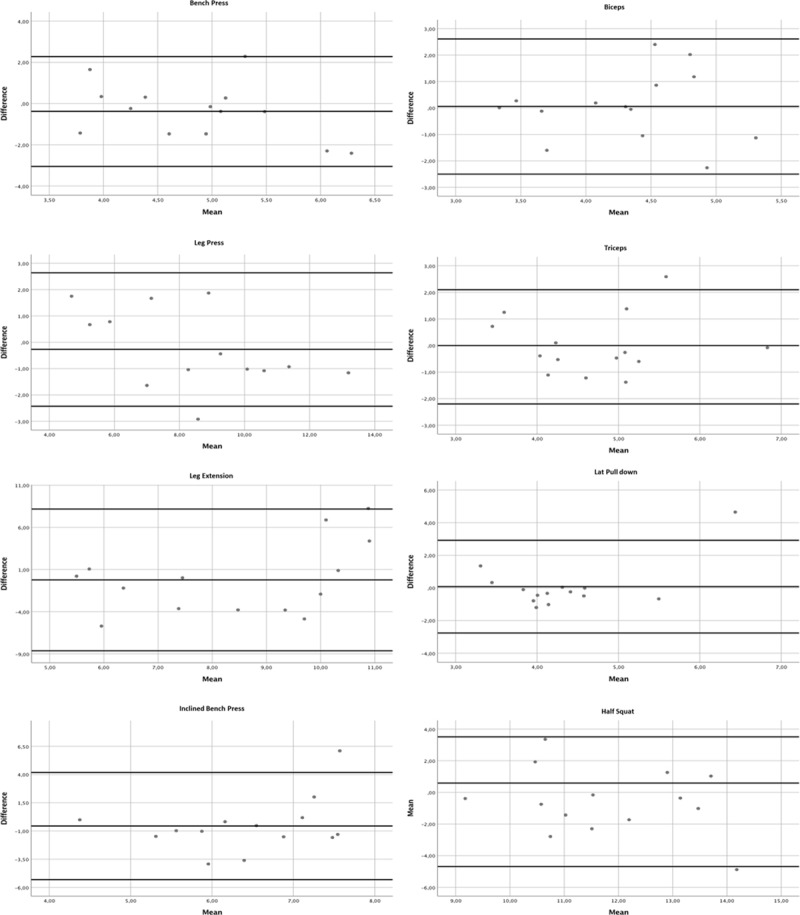
Bland–Altman plots showing the difference between measured and predicted energy cost against the mean of differences in the eight resistance exercises.

Linear regressions between heart rate and energy cost were all significant and presented mean errors below 0.6 kcal·min^-1^ in every exercise (Fig 1). Relative errors, when expressed to the measured energy cost at 24% 1-RM exercise intensity, were all up to 10% (Table 1). Differences between estimated and measured energy cost at 24% 1-RM ranged between -0.08 and +0.59 kcal·min^-1^ and these were all non-significant. Bland-Altman plots of the differences between measured and estimated energy cost confirmed the agreement between the two entities in the eight resistance exercises (Fig 2).

**Table 1:** Prediction equation, adjusted coefficient of determination (ad R^2^), standard error of the regression (S*y*.*x*) and relative error for energy cost prediction at 24% 1-RM in the eight exercises.

	Equation	ad R^2^	S*y*.*x* (kcal·min^-1^)	Error (%)
Triceps extension	EC = 0.0574*heart rate– 2.368	0.994	0.08	1.7
Biceps curl	EC = 0.065*heart rate– 2.845	0.978	0.13	3.0
Half squat	EC = 0.1319*heart rate– 7.780	0.986	0.48	4.1
Leg press	EC = 0.1384*heart rate– 8.097	0.969	0.54	5.2
Leg extension	EC = 0.0976*heart rate– 5.147	0.945	0.59	7.1
Lat pull down	EC = 0.0592*heart rate– 2.458	0.902	0.28	6.4
I Bench press	EC = 0.0694*heart rate– 2.837	0.897	0.54	8.8
H Bench press	EC = 0.0694*heart rate– 2.783	0.814	0.47	10.0

EC = energy cost in kcal·min^-1^; P<0.01 in every regression.

## Discussion

The aim of the present study was to assess the accuracy of heart rate to estimate energy cost during eight resistance exercises performed at low intensities: half squat, 45° inclined leg press, leg extension, horizontal bench press, 45° inclined bench press, lat pull down, triceps extension and biceps curl.

We found that the standard errors of the regression between energy cost and heart rate varied between 0,08 kcal·min^-1^in triceps extension exercise and 0,59 kcal·min^-1^ in leg extension exercise. Relative errors fell below 10% in every exercise and they were lower in triceps extension (1.7%), biceps curl (3%), half squat (4.1%) and leg press (5.2%) exercises. Boudreaux et al [3] recently stated that several HR measurement devices were not accurate to predict energy cost during RE as they often present relative errors above 10%. However, they have evaluated RE at an intensity corresponding to 10-RM, which is much higher than that in the present study. Therefore, at lower RE intensities (i.e. below 30% 1-RM) the use of HR seems to be more accurate.

A higher slope of the regression line between energy cost and heart rate means that for a given increase in heart rate (say per beat) the energy cost increases are steeper. Since in the current study the energy cost is a direct conversion of oxygen uptake, this could mean that muscle O_2_ extraction is better (more O_2_ extracted per beat) the steeper the line. Leg exercise (all the three leg exercises herein) showed higher slopes when compared with upper-body exercises (the remaining 5 exercises). So, an apparent better muscle O_2_ extraction has possibly occurred in leg exercise. This could result from a larger muscle mass involvement in these exercises. A larger muscle mass with a concomitant larger cardiac output may serve to optimize O_2_ delivery and extraction. On the contrary, in small-muscle exercises (such as the biceps curl or triceps extension), despite a lower O_2_ requirements, cardiac output may not suffice to optimize O_2_ delivery and thereby may somehow impair local O_2_ extraction. In fact, the upper-body has a higher proportion of fast-twitch fibres [14], being these related with an increased inefficiency compared with lower body-exercise [15].

Interestingly, the smaller muscle groups showed the lowest standard errors of the regression (biceps and triceps), falling down to 3% relative imprecision. This was somewhat surprising, as the smaller muscle mass induces a low cardiovascular load (as discussed above), which could impair the typical VO_2_ and HR linear relationship. Indeed, it has been shown that the VO_2_ and HR linear relationship is modified according to different muscle masses or modes of exercise. [16] Half squat and leg press, the two exercises involving the largest muscle mass herein, showed also low relative errors (below 5.2%). It could be argued that single-joint exercises (biceps and triceps) could provide a better linearity of the VO_2_ and HR relationship. However, other single-joint exercise such as leg extension showed higher relative error, at 7.1%. Our errors of measurement fall within those typically reported for HR to predict energy cost during cycling and dynamic field leg exercise, between 3 and 12% expressed relative to VO2max. [17]. The absolute errors in the present study averaged 0,4 kcal·min^-1^ (with a maximum of 0,59 kcal·min^-1^), which cam be considered low. Often the literature presents clearly larger errors for HR to predict energy cost at running and cycling exercise, i.e. above 2 kcal·min^-1^ [1]

A possible limitation in the current study is the lack of anaerobic energy measures, such as blood lactate. However, the low-intensity steady-state exercise is consistent with the assumption that steady-state VO_2_ represents overall energy cost of the task. Moreover, blood lactate in resistance exercise performed at intensities below 30% 1-RM fell below the 4 mMol threshold [5,6]. This means that even if blood lactate would have attained 3.5 mMol and taking a realistic pre-exercise value of 2 mMol, lactate accumulation would then be below 2 mMol during a 4-min exercise. This would have an energy equivalent of mere 1.5 ml O_2_/kg/min, a value which would be less than 10% of the energy cost measured solely through VO_2_ at the 24% 1-RM exercise. In leg exercise herein, this fraction would fall below 5%. Hence, it is our belief that this limitation must have not affected our conclusions. Another possible limitation is the absence of a validation group. The reliability of assessing aerobic energy cost through VO_2_ measurements is universally accepted and estimations from anaerobic sources are those who usually include a larger variation. Therefore, we also belief that this limitation does not impair the results herein, although we acknowledge that future confirmation studies are warranted.

## Conclusions

The results herein suggest a potential of wearable heart rate monitors to estimate low-intensity resistance exercises´ aerobic energy cost with errors below 10% in healthy young trained males. The absolute errors of estimate herein are low and acceptable within the framework of exercise prescription. An average error of 0,5 kcal·min^-1^ in a resistance exercise training session performed with low loads (below 30% 1-RM) would results in a mere 15 kcal deviation during a 30 min of amounted workout. Future studies are warranted to confirm the suggestion herein.

## Supporting information

S1 Dataset Measures of energy cost and heart rate in every exercise as well as estimated energy cost for the highest exercise intensity. (XLSX)

## References

[1] Crouter SE, Albright C, Bassett DR Jr. Accuracy of polar S410 heart rate monitor to estimate energy cost of exercise. Med Sci Sports Exerc 2004; 36:1433–1439. 15292754 10.1249/01.mss.0000135794.01507.48

[2] Collins MA, Cureton KJ, Hill DW, Ray CA. Relationship of heart rate to oxygen uptake during weight lifting exercise. Med Sci Sports Exerc 1991; 23: 636–640. 2072844

[3] Boudreaux BD, Hebert EP, Hollander DB, Williams BM, Cormier CL, Naquin MR et al Validity of Wearable Activity Monitors during Cycling and Resistance Exercise. Med Sci Sports Exerc 2018; 50: 624–633. 10.1249/MSS.0000000000001471 29189666

[4] Reis VM, Garrido N, Vianna J, Sousa AC, Vilaça-Alves J, Marques M. Energy cost of isolated resistance exercises across low- to high-intensities. PLoS One 2017; 12: e0181311 10.1371/journal.pone.0181311 28742112 PMC5524349

[5] Oliveira JC, Baldissera V, Simões HG, Aguiar AP, Azevedo P, Poian P et al Identification of the lactate threshold and the blood glucose threshold in resistance exercise. Braz J Sports Med. 2006; 12: 333–338.

[6] Rocha RM, Bomfim DL, Nascimento TB, Moreira SR, Simões HG. Variation in the incremental workload method does not change the lactate threshold determination in resistance exercise. Braz J Sports Med. 2010; 16: 282–285.

[7] American College of Sports Medicine. Position stand: Quantity and quality of exercise for developing and maintaining cardiorespiratory, musculoskeletal, and neuromotor fitness in apparently healthy adults: Guidance for prescribing exercise. Med Sci Sports Exerc. 2011; 43: 1334–1359. 10.1249/MSS.0b013e318213fefb 21694556

[8] Jackson AS, Pollock ML. Generalized equations for predicting body density of men. Br J Nutr. 1978; 40: 497–504. 10.1079/bjn19780152 718832

[9] Kraemer WJ, Fry AC. Strength testing: development and evaluation of methodology In: Maud P and Nieman DC, editors. Fitness and sports medicine: a health-related approach. Palo Alto: Bull Publishing; 1995 pp. 115–138.

[10] Buitrago S, Wirtz N, Yue Z, Kleinoder H, Mester J. Mechanical load and physiological responses of four different resistance training methods in bench press exercise. J Strength Cond Res. 2013; 27: 1091–1100. 10.1519/JSC.0b013e318260ec77 22692106

[11] McLaughlin JE, King GA, Howley ET, Basset DR Jr, Ainsworth BE. Validation of the COSMED K4 b2 portable metabolic system. Int J Sports Med. 2001; 22: 280–284. 10.1055/s-2001-13816 11414671

[12] Welch WA, Strath SJ, Swartz AM. Congruent validity and reliability of two metabolic systems to measure resting metabolic rate. Int J Sports Med. 2015; 36: 414–418. 10.1055/s-0034-1398575 25700097

[13] Tian Y, He Z, Xu C, Huang C, Lee JH, Li R, et al Energy expenditure and fitness responses following once weekly hill climbing at low altitude. Int J Sports Med. 2015; 36: 357–364. 10.1055/s-0034-1395520 25607522

[14] Bernasconi S, Tordi N, Perrey S, Parratte B, Monnier G. Is the VO2 slow component in heavy arm-cranking exercise associated with recruitment of type II muscle fibers as assessed by an increase in surface EMG? Appl Physiol Nutr Metab. 2006; 31: 414–422. 10.1139/h06-021 16900231

[15] Koppo K, Bouckaert J, Jones AM. Oxygen uptake kinetics during high-intensity arm and leg exercise. Respir Physiol Neurobiol. 2002;133: 241–250. 12425971 10.1016/s1569-9048(02)00184-2

[16] Rayson MP, Davies A, Bell DG, Rhodes-James ES. Heart rate and oxygen uptake relationship: a comparison of loaded marching and running in women, Eur J Appl Physiol 1995; 71: 405–408. 10.1007/BF00635873 8565971

[17] Bot SD, Hollander AP. The relationship between heart rate and oxygen uptake during non-steady state exercise. Ergonomics 2000; 43: 1578–1592. 10.1080/001401300750004005 11083138

