# Correction: Are wearable heart rate measurements accurate to estimate aerobic energy cost during low-intensity resistance exercise?

**DOI:** 10.1371/journal.pone.0230323

**Published:** 2020-03-05

**Authors:** Victor M. Reis, Jeferson M. Vianna, Tiago M. Barbosa, Nuno Garrido, Jose Vilaça Alves, André L. Carneiro, Felipe J. Aidar, Jefferson Novaes

There are errors in the Funding statement. The correct Funding statement is as follows: This research was supported by national funds through FCT (Fundação para a Ciência e Tecnologia) under the project UID/DTP/04045/2019. The funders had no role in study design, data collection and analysis, decision to publish, or preparation of the manuscript.
